# Tool for tracking all-cause mortality and estimating excess mortality to support the COVID-19 pandemic response

**DOI:** 10.5365/wpsar.2022.13.2.921

**Published:** 2022-05-25

**Authors:** Duan Mengjuan, Mark S. Handcock, Bart Blackburn, Fiona Kee, Viema Biaukula, Tamano Matsui, Babatunde Olowokure

**Affiliations:** a Division of Data, Strategy & Innovation Team, World Health Organization Regional Office for the Western Pacific, Manila, Philippines.; b Department of Statistics, University of California, Los Angeles, CA, United States of America.; c Division of Health Security and Emergencies, World Health Organization Regional Office for the Western Pacific, Manila, Philippines.

## Abstract

**Problem:**

Quantifying mortality from coronavirus disease (COVID-19) is difficult, especially in countries with limited resources. Comparing mortality data between countries is also challenging, owing to differences in methods for reporting mortality.

**Context:**

Tracking all-cause mortality (ACM) and comparing it with expected ACM from pre-pandemic data can provide an estimate of the overall burden of mortality related to the COVID-19 pandemic and support public health decision-making. This study validated an ACM calculator to estimate excess mortality during the COVID-19 pandemic.

**Action:**

The ACM calculator was developed as a tool for computing expected ACM and excess mortality at national and subnational levels. It was developed using R statistical software, was based on a previously described model that used non-parametric negative binomial regression and was piloted in several countries. Goodness-of-fit was validated by forecasting 2019 mortality from 2015–2018 data.

**Outcome:**

Three key lessons were identified from piloting the tool: using the calculator to compare reported provisional ACM with expected ACM can avoid potential false conclusions from comparing with historical averages alone; using disaggregated data at the subnational level can detect excess mortality by avoiding dilution of total numbers at the national level; and interpretation of results should consider system-related performance indicators.

**Discussion:**

Timely tracking of ACM to estimate excess mortality is important for the response to COVID-19. The calculator can provide countries with a way to analyse and visualize ACM and excess mortality at national and subnational levels.

## PROBLEM

Coronavirus disease 2019 (COVID-19), caused by severe acute respiratory syndrome coronavirus 2 (SARS-CoV-2), was identified in late December 2019 and declared a pandemic by the World Health Organization (WHO) on  11 March 2020. (*1*) In the WHO Western Pacific Region, by the end of November 2021, there were 10 221 280 confirmed COVID-19 cases and 141 864 deaths. (*2*) Although the COVID-19 death count is essential to understanding the epidemiology of COVID-19, the attributable mortality due to COVID-19 remains unclear. In any given country, official statistics may not reflect the actual number of lives lost to the disease. (*3*)

Identifying deaths from COVID-19 is difficult, especially in low-resource settings. (*4*) Many countries have limited capacity for COVID-19 testing at national and subnational levels, and therefore no capability to track the spread of COVID-19. Even where cases are adequately detected, some deaths may not be reported promptly or even at all. (*4*) Also, reporting of cause of death may be inaccurate because the quality of death certification depends on the knowledge and skills of physicians, on the characteristics of the deceased person (older people are the most difficult to certify correctly), on errors in coding the death event and on the format of certification. (*5*) There can also be a long lag between the death occurring and being certified, especially for deaths outside hospitals or other health-care facilities, or those that require an autopsy. Service interruptions due to the pandemic may further delay the death certification process.

According to an internal rapid assessment in the WHO Western Pacific Region, most Member States have two to four death reporting systems. Most systems are electronic or partially electronic, and although some are well integrated within civil registration and vital statistics systems, others are disjointed. The United Nations Statistics Division estimated that death registration coverage is over 80% in 15 of the 27 Western Pacific Regional Member States with data available. (*6*) Total death counts, reported either weekly or monthly, are publicly available from at least six Member States, and data are available internally from at least four. Thus, it may be feasible for several Member States in the WHO Western Pacific Region to track all-cause mortality (ACM) to provide timely information on COVID-19 deaths. Ideally, deaths would be reported as soon as possible, with more detailed information (e.g. cause of death) reported later when death certificates become available.

## CONTEXT

Tracking current ACM and comparing it with expected ACM from pre-pandemic data can provide an estimation of the overall burden of mortality potentially related to the COVID-19 pandemic. (*4*) This method requires first estimating the number of deaths that would be expected if the COVID-19 pandemic had not occurred (i.e. expected deaths) using historical data and a sophisticated statistical model. (*7*) Excess mortality is then estimated by comparing the current reported provisional deaths with the expected deaths. (*8*)

The excess mortality may be directly or indirectly due to COVID-19. Indirect deaths due to COVID-19 include those linked to conditions that were present before the pandemic and have resulted in death because health systems were overwhelmed, those due to patients avoiding health-care facilities and those linked to routine service delivery interruption for non-COVID-19 disease. These indirect deaths due to COVID-19 are not captured in the COVID-19 death numbers reported to WHO. (*9*) Given that COVID-19 deaths can influence national and subnational response measures, additional effort is required to ensure that this information is readily available and quickly tracked.

A common method to estimate the expected ACM is to use the average death count for each week over a 5-year period. However, this method does not account for the seasonality of mortality, or for the trend and smoothness of expected mortality from week to week or month to month. Additionally, if a trend is present over time, using historical averages will not capture the trend or allow it to be projected into the future. A more sophisticated method by Weinberger et al. (*10*) fits Poisson regression models that adjust for seasonality, year-to-year baseline variation, influenza epidemics and reporting delays. Our statistical model, the WHO Western Pacific Regional Office ACM calculator (hereafter, the ACM calculator), is based on this method.

## ACTION

### The WHO Western Pacific Regional Office ACM calculator

The ACM calculator was developed to assist Member States in the WHO Western Pacific Region in tracking and analysing ACM. (*11*) The user enters the relevant ACM data into the designated template in the calculator, and the expected ACM and excess mortality are calculated.

The calculator can be used online or installed onto a local machine. The input data are never stored offline and are only accessible to the user. Depending on the amount of data entered, the calculator will finish computing within seconds or minutes. Various outputs are available, including disaggregated results; for example, the calculator can provide expected ACM by age group and sex if the data entered are disaggregated by these factors. The results can be displayed in a variety of formats, including tables and graphs. (*11*)

### Statistical methods

The ACM calculator is based on the model of Weinberger et al., (*10*) but uses non-parametric negative binomial regression. This approach was preferred to Poisson regression because it allows for overdispersion and can account for instances of low or zero counts. (*10*) The mean function includes a smooth trend and a smooth non-parametric annual cycle in mortality over time. These terms were specified using cubic smoothing splines, including a cyclical one for the annual cycle. The model allows for arbitrary time-varying covariates, and the parameters were estimated through restricted maximum likelihood estimation. The methodology does not currently adjust for influenza epidemics and reporting delays because this information is not consistently reported.

The expected ACM deaths are forecast stochastically, to represent uncertainty in the estimate of the expected. Thus, statistical significance in observed data can be determined (i.e. a substantial increase or decrease from the baseline). The forecast is an average over the sampling distribution of the parameter estimates, which is a simple way to account for uncertainty in the expected deaths, in addition to the sampling variation of the counts for given model parameters. This approach is preferred to a formal Bayesian model because of its simplicity. The model goodness-of-fit was validated by forecasting 2019 mortality from 2015–2018 data (see **Appendix**

for details). The validation indicated that the statistical coverage of the procedure is close to its nominal rate and that the prediction interval lengths are smaller than those based on the historical average model. The intervals based on the historical average are misleading and their actual coverage is far below their nominal coverage.

The calculator was developed using R statistical software (ver. 4.1.2), which includes the estimation of historical patterns and the computation of expected ACM. The software computes the excess mortality from 2020 to the present time; displays different visualizations of expected ACM and excess mortality and allows these visualizations and their raw data to be downloaded for further analysis and inclusion in reports; and includes interactive help and documentation of the methodology. The software is open-source. For reproducibility purposes, the exact code used for the analyses in this paper is in a static archive. (*12*)

## OUTCOME

The ACM calculator has been tested using publicly available data from several Member States. Two examples are provided to highlight key lessons from implementing the calculator.

The first example from one country (January through September 2020) compares ACM plots using the calculator versus ACM plots based on historical averages only. The results from the calculator showed that the recorded counts were well within the 95% prediction interval generated (**Fig. 1A**). Although the reported counts were sometimes above the expected counts (most notably in August), the reported counts were always within the prediction interval. In contrast, the recorded counts based on the historical average only were well above the historical average (**Fig. 1B**) but confidence intervals and statistical increase were not calculated. The calculator values are above the historical average because of the presence of an upward trend in reported counts from 2015 to 2019; the calculator takes this into account whereas the historical average does not. Because historical averages do a poor job of predicting, comparison with the monthly average alone would lead to false conclusions.

**Figure 1A:**
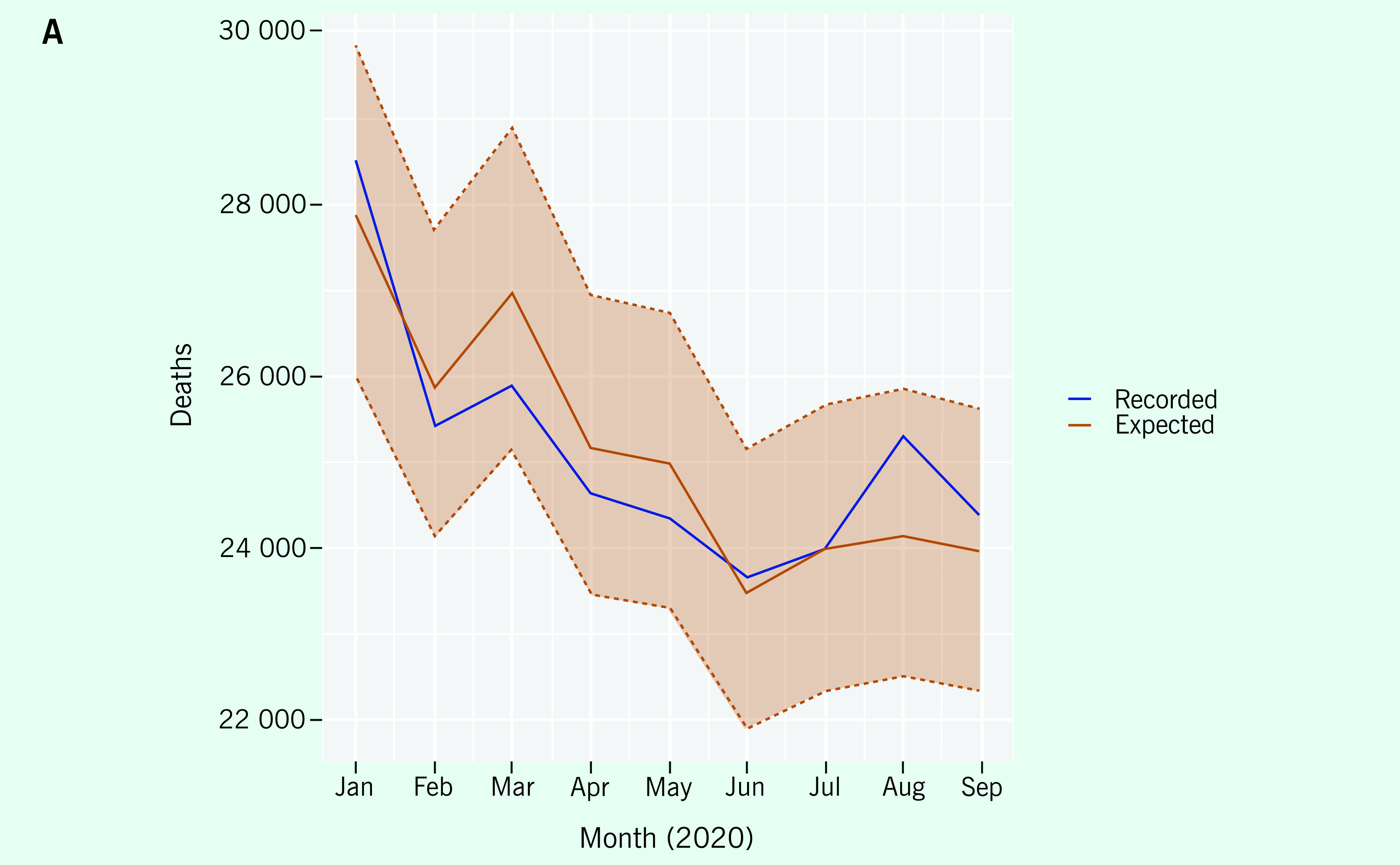
A) Monthly reported ACM compared with expected ACM for the first 9 months of 2020 using the calculator. The red zone is the 95% prediction interval. B) Monthly reported ACM compared with theexpected ACM and the historical average ACM. The blue lines plot the recorded number of deaths, the orange the expected number of deaths under the model and the green the average number of deaths by month during 2015–2019.

[**insert** Figure 1A]

**Figure 1B:**
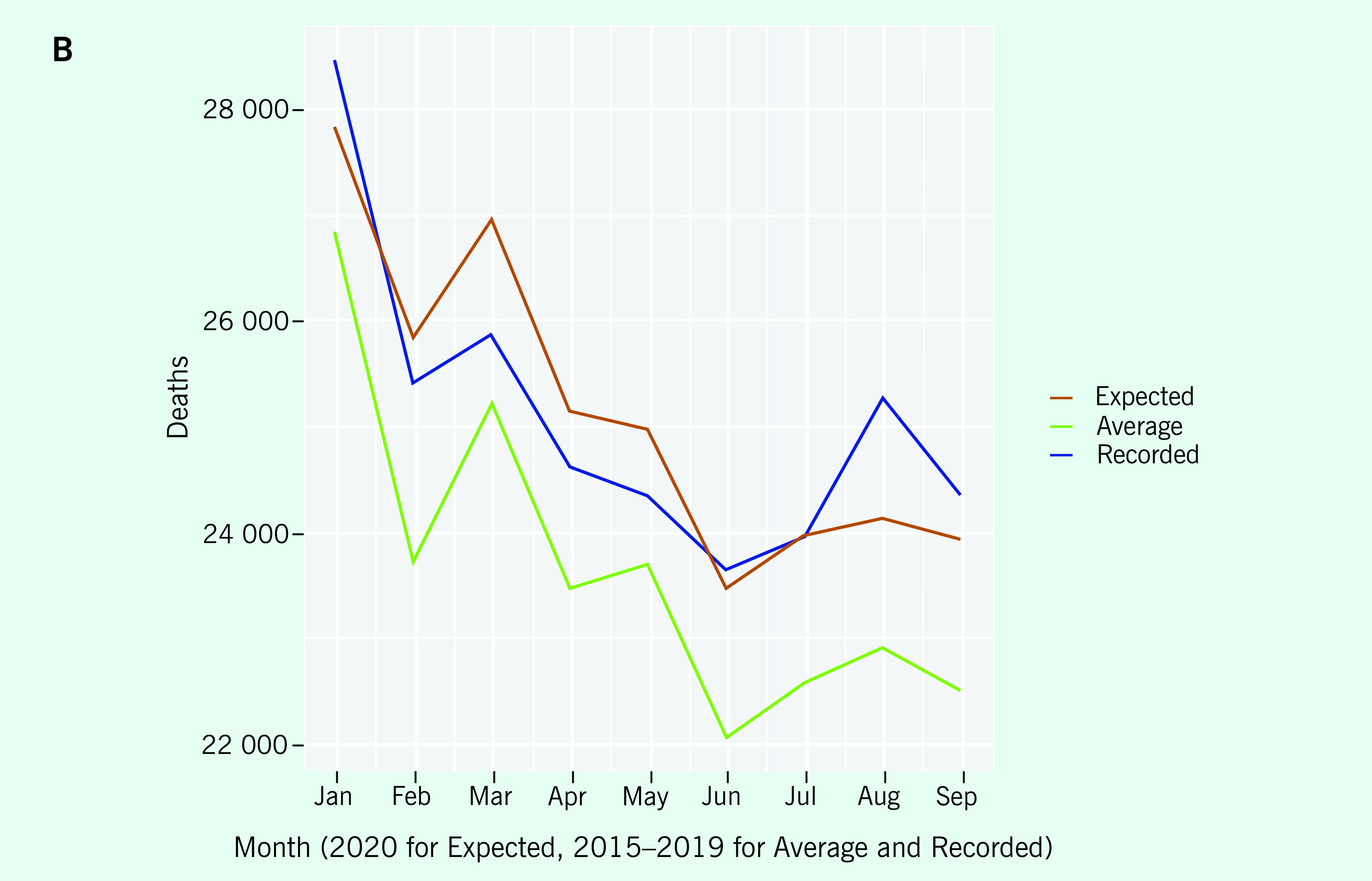
A) Monthly reported ACM compared with expected ACM for the first 9 months of 2020 using the calculator. The red zone is the 95% prediction interval. B) Monthly reported ACM compared with theexpected ACM and the historical average ACM. The blue lines plot the recorded number of deaths, the orange the expected number of deaths under the model and the green the average number of deaths by month during 2015–2019.

[**insert** Figure 1B]

The second example illustrates the ability of the calculator to show hidden excess mortality within subregions based on disaggregated data. Using data from another country, the national data indicate no excess mortality over a particular period (**Fig. 2A**), whereas the data for that period from a single local region show excess mortality during July and August that is outside the 95% prediction intervals for these months  (**Fig. 2B**). Therefore, the excess mortality for July and August is statistically significantly different from zero (even after adjusting for multiple comparisons). (*7*) This example highlights the value of being able to analyse subregions, because excess mortality may not be identifiable at the national level in some cases.

**Figure 2A:**
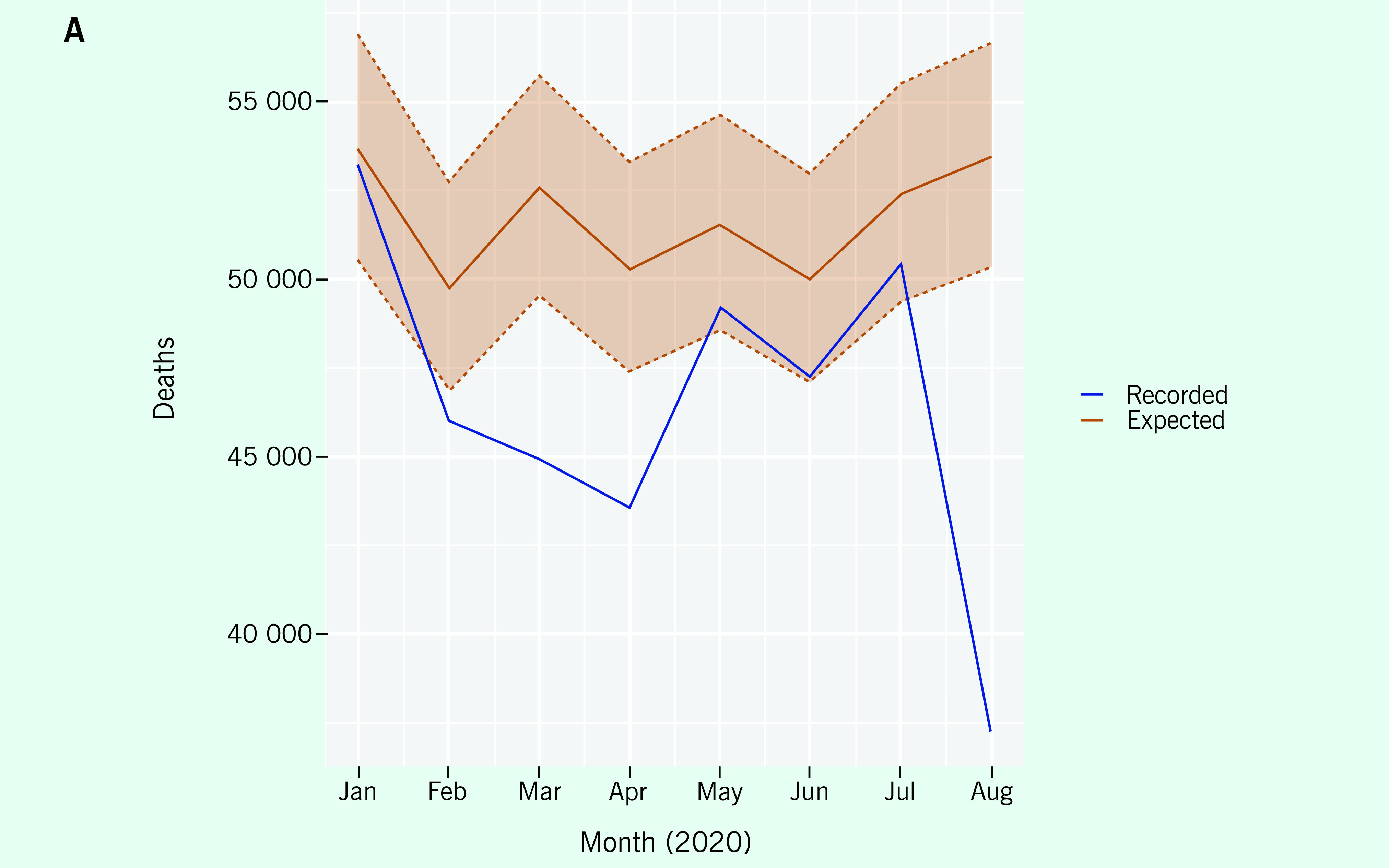
ACM at the national (A) and subregional level (B) within the same member state in the WHO Western Pacific Region. Looking at the aggregate would lead to a conclusion of no excess mortality present; however, by disaggregating the data into subregions we can identify areas where significant excess mortality is present.

[**insert** Figure 2A]

**Figure 2B:**
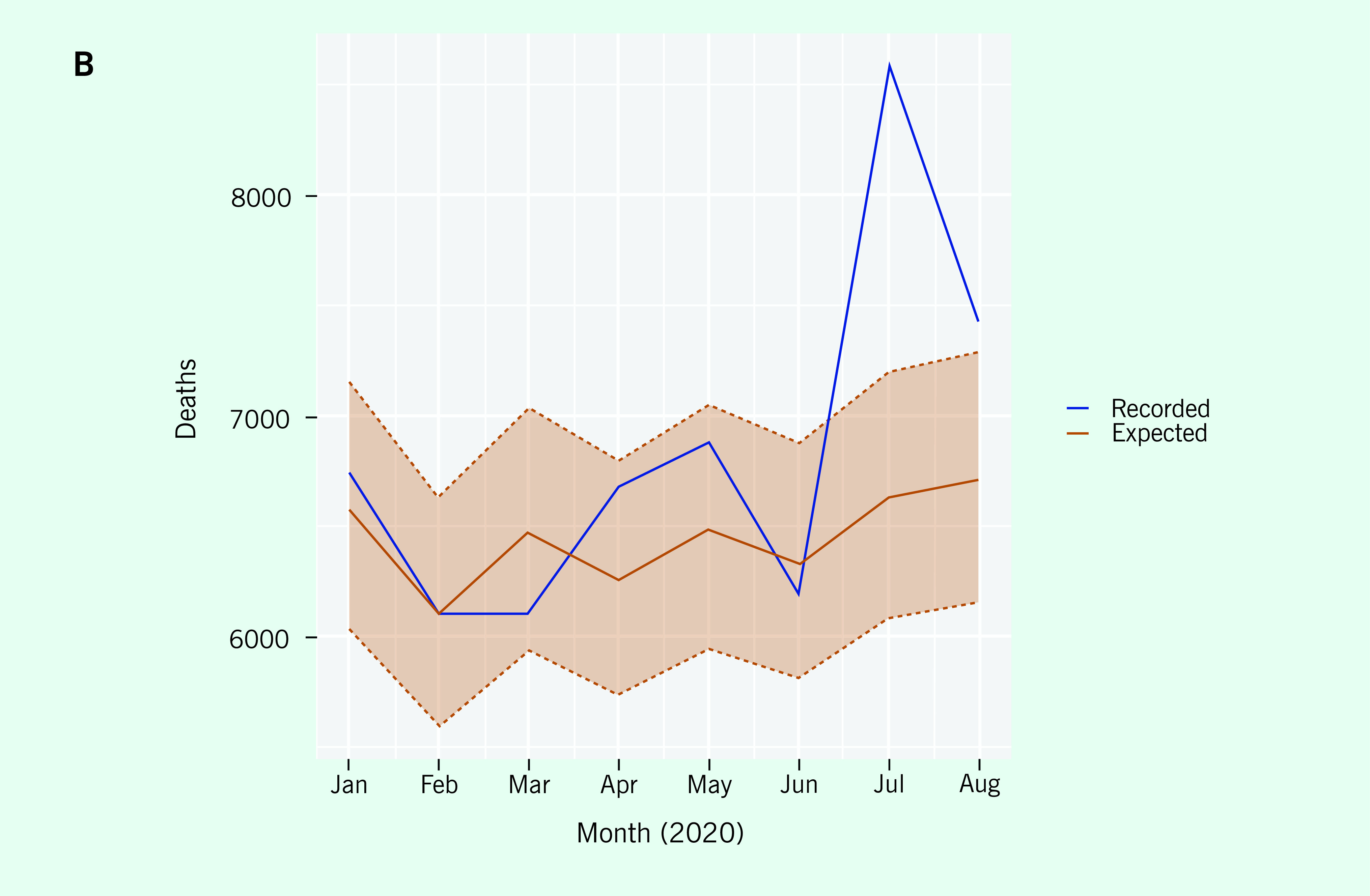
A) Monthly reported ACM compared with expected ACM for the first 9 months of 2020 using the calculator. The red zone is the 95% prediction interval. B) Monthly reported ACM compared with theexpected ACM and the historical average ACM. The blue lines plot the recorded number of deaths, the orange the expected number of deaths under the model and the green the average number of deaths by month during 2015–2019.

[**insert** Figure 2B]

### Lessons identified

Three key lessons were identified from piloting the tool: using the calculator to compare reported provisional ACM with expected ACM can avoid potential false conclusions from comparing with historical averages alone; using disaggregated data at the subnational level (e.g. by region, sex and age) can detect excess mortality by avoiding dilution of total numbers at the national level; and interpretation of results should consider system-related performance indicators such as system coverage, completeness and reporting delays.

### Suggestions for interpreting results

Given that the quality of mortality reporting varies greatly within and between Member States, the results of the ACM calculator should be interpreted with caution. Death coverage may differ if mortality reporting systems do not cover all death counts, with inconsistencies if a country has multiple systems, especially in low-resource settings. Civil registration of deaths is often below 20% in low- and middle-income countries. (*4*) There are also timeliness issues and reporting delays, so the death count may be incomplete for certain periods (e.g. the latest week or month). It can take more than 12 months for mortality data to be finalized at the national level owing to deaths not being promptly reported or registered by subnational authorities, a long lag between a death and completion of the death certificate, a backlog at the subnational level that delays reporting to the national level and long processing times for the reporting systems. The use of disaggregated data to improve monitoring sensitivity may be affected by differences in the severity of COVID-19 transmission between subnational regions; also, the impact may vary among different population groups (e.g. by sex, age and occupation).

Proactively tracking ACM at the local level may help to capture more timely information, given that reporting and validation from the local to the national level may take several months to complete. Also, in both the short and long-term, careful interpretation of the results is crucial to tailor specific actions based on conditions within each Member State.

For countries with existing systems that cover compulsory and universal mortality reporting, it is important to make use of the existing data to monitor weekly and monthly trends, to drive decision-making. For countries with low levels of mortality reporting coverage, it is still worth monitoring weekly and monthly trends based on available data; however, results should be interpreted with caution, as mentioned above. Additional resources or channels (e.g. burial or cemetery registration) can be employed to track total death counts. Community based mortality reporting should also be considered if necessary.

### Limitations

There are two main limitations to the calculator. First, our methodology assumes that reported counts are the actual values and that reports are complete and accurate. However, provisional death counts are normally used for timely monitoring. Results should be compared with in-place systems, as mentioned above. Second, the fundamental assumption is that the statistical variation in ACM during the historical period (2015–2019) is the same as that from 1 January 2020 onward in the counter-factual situation where there was no pandemic. This is not directly testable because of confounding by the pandemic itself. In addition, it is assumed that the negative binomial regression model is adequate to capture this variation, and that counts are independent from period to period (conditional on the annual cycle and covariates). If these assumptions are incorrect, the estimates and prediction intervals will be inaccurate and probably overly optimistic.

## Discussion

During an epidemic or other public health emergency where mortality occurs, such as the COVID-19 pandemic, many countries experience disruption to routine health-care services and socio-behavioural changes in the population. For example, 90% of countries have reported disruptions to essential health services since the COVID-19 pandemic began. (*13*) These changes, together with a lack of reliable data and reporting systems, make the true burden of the pandemic difficult to quantify. ACM, when reported in a timely manner, can be used to estimate excess mortality, providing a rapid snapshot of the situation to support decision-makers to identify the extent and progression of the pandemic. Analysing and interpreting ACM data (including disaggregated data) can also provide important information about who is dying and where, which can then guide decisions on targeted surveillance and efficient use of health resources. The ACM calculator was developed to make it easy for Member States to analyse and visualize their ACM data. Users reported that the tool allowed them to analyse data on their own and easily generate results. Although the underlying statistical model is sophisticated, the use of complex algorithms in the background provides state-of-the-art summaries in the foreground. The model is standardized for a broad user base but could be customized for the needs of specific Member States. However, caution should be exercised when interpreting the results.
