# Supplementary material for: Tool for tracking all-cause mortality and estimating excess mortality to support the COVID-19 pandemic response
Source: Western Pac Surveill Response J. 2022 May 25;13(2):1–7. doi: 10.5365/wpsar.2022.13.2.921 (PMC9580363; doi:10.5365/wpsar.2022.13.2.921)
Supplement: Supplementary file 1 [file wpsar-13-921-s001.pdf]

APPENDIX

This appendix provides technical details on the statistical methods used to estimate excess mortality during the coronavirus disease (COVID-19) pandemic using data on all-cause mortality (ACM). It also provides a simulation study to assess the validity of the methodology. The exact code used in the analyses in this paper is in a static archive.[1]

We consider the case where we have multiple time-series of ACM counts from each Member State for each week between 1 January 2015 and a recent date. For some Member States we have only monthly data; for such cases, the description below is also suitable. We consider the case where we have separate reported counts for each sex and age group (typically, 5-year age groups).

The primary objective is to estimate the expected ACM counts for each week from 1 January 2020 onward assuming no pandemic had occurred. The excess mortality is defined as the difference between the reported counts and expected counts for each week.

Model

To illustrate, let us consider the case of females aged 65–74 years in Australia. Let y_t be the count for week t=1,…,T with t=1,…,260 being the period 1 January 2015 to 31 December 2020. We model y_t as a random variable following a negative-binomial distribution with mean parameter λ_t. We make this choice rather than using a Poisson distribution to account for overdispersion in the counts. The overdispersion parameter is itself estimated from the data and the mean parameters λ_t are modelled as:

$$\log \lambda_t = c(t) + trend(t) + X_t\beta$$

where $c(t)$ represents the annual cycle in ACM and $trend(t)$ is the curvilinear trend of ACM over time. The annual cycle $c(t)$ is modelled as a cyclic cubic spline function[2] of time with a period of 52 weeks (i.e. $c(t) = c(t + 52)$), where a spline is a piecewise polynomial. Conceptually, one can imagine a high-degree polynomial capable of crossing through every data point. Such a polynomial would probably overfit the observed data, meaning it may not predict well using new data. Splines allow many low-degree (in this case, degree three) polynomials to fit the data in pieces, achieving a good fit to the data without the risk of overfitting.

Specifically, c_t is modelled as a piecewise cubic polynomial that has a continuous second derivative, is continuous, has continuous first and second derivatives at 52-week cycles and best fits the recorded ACM while being smooth. The specific criterion for the last feature is to choose c_t to minimize the penalized square error (PSE):

$$PSE_\tau(c) = \text{log-restricted-likelihood}\,(y, X, t = 1, \dots, T) - \tau \int_0^{52} c''[s]^2\, ds \quad \tau > 0$$

where $c''[s]$ is the second derivative of $c[s]$ and τ is a smoothing parameter, chosen to balance the closeness of fit to the recorded counts (the first term) with the smoothness of $c[s]$ (the second term). Hence, choosing the function $c[s]$ that minimizes $PSE_\tau(c)$ provides a balanced representation of the annual cycle. It prioritizes smoothness of $c[s]$ over the closeness of fit of $c[s]$ to the recorded ACM. The traditional estimator, $c[s]$, is the minimizer with τ=0; that is, there is no penalty for lack of smoothness. The choice of τ is subjective. In this work we chose to maximize the ability to predict unrecorded ACM counts. Specifically, we used generalized cross validation (GCV)[3] to choose, and the R package 'mgcv' (created by Simon Wood) for analysis.[4,5] The annual cycle obtained in this way is the optimal smoothest annual cycle chosen to maximize the likelihood of the observed ACM.

A similar approach is taken to the curvilinear trend $trend(t)$. It is modelled as a (non-cyclic) cubic spline function – specifically, as a piecewise cubic polynomial that has a continuous second derivative, is continuous and best fits the recorded ACM while being smooth. The specific criterion for the last feature is to choose $trend(t)$ to minimize the PSE:

$$PSE_\gamma(trend) = \text{log-restricted-likelihood}\,(y, X, t = 1, \dots, T) + \gamma \int_0^{260} trend''[t]^2\, dt \quad \gamma > 0$$

where $trend''[t]$ is the second derivative of $trend(t)$ and γ is a smoothing parameter, chosen to balance the closeness of fit to the recorded counts (the first term) with the smoothness of $trend(t)$ (the second term). Hence, choosing the function $trend(t)$ that minimizes PSE_{γ} $(trend)$ provides a balanced representation of the trend. It prioritizes smoothness of $trend(t)$ over the closeness of fit of $trend(t)$ to the recorded ACM. The traditional estimator, $trend(t)$, is the minimizer with $\gamma=0$; that is, there is no penalty for lack of smoothness. Like τ, the choice of γ is subjective. Also, as with the annual cycle, we chose to maximize the ability to predict unrecorded ACM counts by using the GCV criterion. The model allows for arbitrary time-varying covariates, X_t. Including both the date and period allows for the model to detect trends both across and within years.

Negative-binomial regression is a natural choice given that we are seeking to estimate the death count during any time frame. Negative-binomial is preferred to Poisson regression because it allows for overdispersion; also, it can account for instances of low or zero counts without issue.

This particular negative-binomial regression model is a generalized additive model (GAM) that uses smoothing functions for the predictor variables. Since the date and period are input as discrete values, they are smoothed using cubic splines, a common smoothing technique. The parameters β and the splines themselves are found through restricted maximum likelihood estimation. GAMs are a type of generalized linear model, which are generalizations of ordinary linear regression that allow for the response variable to have error distributions other than the normal distribution (in this case, the negative-binomial distribution).

Currently, this model is simple in that it uses only information on sex, age group and time/date. When more data become readily available (e.g. influenza counts), the model can easily be extended to incorporate that data. There are also other ways to enhance the model, such as considering negative-binomial regression for the case of overdispersion or using hierarchical models for sharing information across groupings. Hence, this preliminary approach should serve as a strong starting point.

The next step is to stochastically forecast the expected to represent the uncertainty in the estimate of the expected. Thus, the statistical significance of the observed can be determined (i.e. if it represents a substantial increase or decrease from the baseline). One detail of the forecast is that it is an average over the sampling distribution of the parameter estimates. This is a simple way to account for uncertainty in our model for the expected mortality in addition to the sampling variation of the counts for given model parameters. We prefer this to a formal Bayesian model owing to its simplicity.

Currently, models are fit separately to each sex, each age group and each Member State. It is possible to improve the estimation by using information from both sexes and multiple age groups simultaneously, but this is a bias–variance trade-off that can be explored.

For Member States with missing (pandemic) weeks, we can stochastically interpolate using simple time-series models. If the number of missing weeks is significantly high, we use a negative-binomial model such as the one described above to stochastically interpolate.

An issue that may be important to adjust for is reporting delay (this is mainly an issue for recent weeks). To do this, information is needed on the reporting delay. In the United States, the National Center for Health Statistics reports mortality as the serial provisional data from the states are received and processed – counts of deaths from recent weeks are highly incomplete, reflecting delays in reporting. These "provisional" counts are updated regularly over the following weeks, and the counts are not finalized until more than a year later. The estimate of completeness is based on the number of weeks that have passed between when the death occurred and when the data set was obtained. We can model this relationship and use it to adjust the estimates, if necessary.

Validation of the statistical method for estimating ACM without a pandemic

One may ask why it is not better to simply compare the observed ACM counts to historical averages of recent years. As we will show, doing so offers less robust pre-

diction intervals than using the model described above. The following validation metrics also justify using this model to gauge the significance of current ACM counts relative to pre-pandemic times.

The model attempts to forecast ACM counts for each week of 2020 and beyond, assuming no pandemic had occurred. Since the discrepancy between actual counts and expected counts is the sought-after estimate of excess mortality in 2020, it is vital that the model makes accurate predictions. One way to validate the accuracy of the model is to use it to predict ACM during 2019, a year in which there would have been no "excess" mortality. The model is trained using data from 1 January 2015 through to 31 December 2018, then predictions are made on a weekly or monthly basis for 2019. The closer the predicted counts are to the observed counts, the better the model is performing.

The model has been validated across all age groups, sexes and Member States, but to continue with the example used earlier (i.e. of females aged 65–74 years in Australia), we present those results for that example. **Appendix Fig. 1** below shows the 95% prediction intervals for the model ("spline") and for the weekly average. The actual weekly counts are denoted by the black dots, showing that the spline model fails to capture the true count just three times out of 52 periods (95% accurate). The weekly average fairs far worse. As is evident from **Appendix Fig. 1**, the lengths of the spline intervals are typically smaller than the lengths of the weekly average intervals, meaning that the spline

Appendix Fig. 1. **Prediction intervals for 2019 based on deaths in 2015–2018. The black dots are the reported deaths for each week in 2019. The green error bars are based on the weekly averages. The blue intervals are based on the spline model. Those based on the weekly averages are incorrect and their actual coverage is well below their nominal coverage. The intervals based on the spline model are valid.**

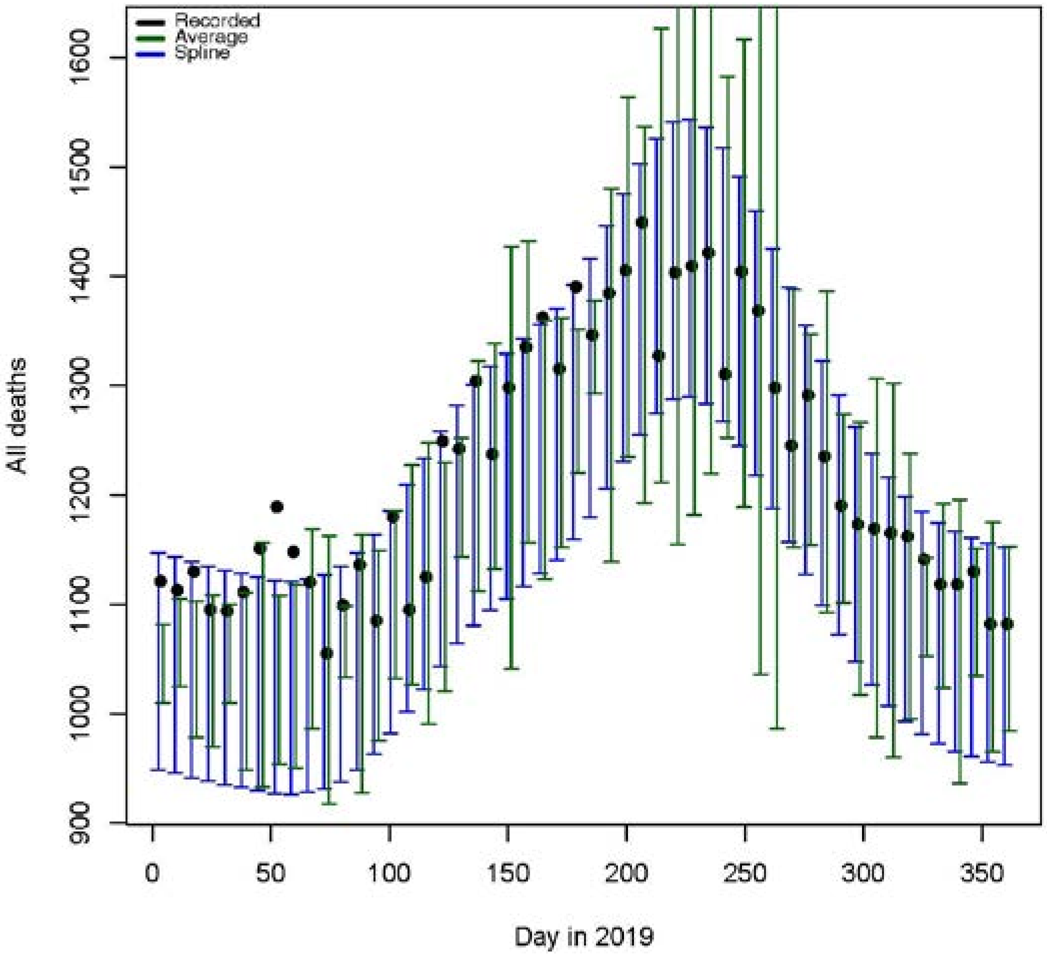

Appendix Table 1. **Prediction interval accuracy for all age and sex groups. The intervals produced by the spline model have the correct coverage whereas those produced by the weekly average model are well below their nominal coverage.**

Sex and age group	Average (PI %)	Spline (PI %)
Female 0–44	85	94
Female 45–64	83	93
Female 65–74	81	91
Female 75–84	92	93
Female ≥85	87	96
**Female total**	88	95
Male 0–44	83	89
Male 45–64	81	97
Male 65–74	92	92
Male 75–84	87	91
Male ≥85	81	87
**Male total**	75	86
Total 0–44	87	89
Total 45–64	85	95
Total 65–74	88	90
Total 75–84	81	92
Total ≥85	81	95
**Overall total**	83	91
**Median %**	84	92
**Mean %**	84	92

ETS: exponential triple smoothing; PI: prediction interval.

model has higher accuracy because it is a better model rather than just because it is larger. More importantly, the weekly average intervals are misleading and their actual coverage is far below their nominal coverage.

The accuracy of the spline model is not solely for females aged 65–74. **Appendix Table 1** shows per cent accuracy (i.e. how often the prediction interval contains the actual value) for each demographic breakdown. The spline model significantly outperforms the weekly average across all sex and age groups.

Another way to check the validity of the model is to look at the length of the prediction intervals. The intervals should be long enough to capture the true values most of the time; however, intervals that are too long create too much uncertainty to be worthwhile. **Appendix Table 2** shows the lengths of the prediction intervals for the spline, exponential triple smoothing (ETS) and weekly average. The spline intervals tend to be nearly the same length as those of the ETS for those aged 0–74 (the weekly average has a short length but is highly inaccurate). It is in those aged 75+ (and when aggregating across all age groups) that the spline intervals are longer than their counterparts. The significant increase in the uncertainty surrounding the older age categories is something that will be investigated.

Appendix Table 2. **Prediction interval length for all Australian age and sex groups.**

Member state: Sex and age group	Average from 2015 to 2018 (PI length)	ETS (PI length)	Spline (PI length)
AUS: Female 0–44	8.9	20	20
AUS: Female 45–64	18.6	43	43
AUS: Female 65–74	24.9	52	53
AUS: Female 75–84	37.8	70	78
AUS: Female ≥85	70.3	103	148
**AUS: Female total**	106.5	143	225
AUS: Male 0–44	10.0	21	21
AUS: Male 45–64	23.6	50	53
AUS: Male 65–74	36.0	63	72
AUS: Male 75–84	45.1	78	93
AUS: Male ≥85	60.2	87	118
**AUS: Male total**	107.2	143	210
AUS: Total 0–44	13.8	29	29
AUS: Total 45–64	28.3	66	66
AUS: Total 65–74	50.8	81	98
AUS: Total 75–84	64.7	105	130
AUS: Total ≥85	111.9	135	228
**AUS: Overall total**	191.0	202	386
**Median length**	41.5	74	85
**Mean length**	56.1	83	115

AUS: Australia; ETS: exponential triple smoothing; PI: prediction interval.

References

1. Handcock MS, Blackburn B. Handcock/WPROACM: Methodology and code used in the WHO WPRO ACM Calculator (v1.0.0). Geneva: Zenodo; 2021. doi:10.5281/zenodo.5746071
2. Wegman EJ, Wright IW. Splines in statistics. J Am Stat Assoc. 1983;78(382):351–65. doi:10.2307/2288640
3. Craven P, Wahba G. Smoothing noisy data with spline functions. Estimating the correct degree of smoothing by the method of generalized cross-validation. Numer Math. 1979;31:377–403.
4. Wood SN. Stable and efficient multiple smoothing parameter estimation for generalized additive models. J Am Stat Assoc. 2004;99(467):673–86. doi:10.1198/016214504000000980
5. Wood SN. Generalized additive models: an introduction with R. 2nd ed. Boca Raton: Chapman and Hall/CRC Press; 2017.
